# An advanced Artificial Intelligence platform for a personalised treatment of Eating Disorders

**DOI:** 10.3389/fpsyt.2024.1414439

**Published:** 2024-08-06

**Authors:** Francesco Monaco, Annarita Vignapiano, Martina Piacente, Claudio Pagano, Carlo Mancuso, Luca Steardo, Alessandra Marenna, Federica Farina, Gianvito Petrillo, Stefano Leo, Emanuela Ferrara, Stefania Palermo, Vassilis Martiadis, Marco Solmi, Alessio Maria Monteleone, Alessio Fasano, Giulio Corrivetti

**Affiliations:** ^1^ Department of Mental Health, Azienda Sanitaria Locale Salerno, Salerno, Italy; ^2^ European Biomedical Research Institute of Salerno (EBRIS), Salerno, Italy; ^3^ Innovation Technology e Sviluppo (I.T.Svil), Salerno, Italy; ^4^ Psychiatric Unit, Department of Health Sciences, University Magna Graecia of Catanzaro, Catanzaro, Italy; ^5^ Department of Mental Health, Azienda Sanitaria Locale (ASL) Napoli 1 Centro, Napoli, Italy; ^6^ Department of Psychiatry, University of Ottawa, Ontario, ON, Canada; ^7^ On Track: The Champlain First Episode Psychosis Program, Department of Mental Health, The Ottawa Hospital, Ontario, ON, Canada; ^8^ Clinical Epidemiology Program, Ottawa Hospital Research Institute, University of Ottawa, Ottawa, ON, Canada; ^9^ School of Epidemiology and Public Health, Faculty of Medicine, University of Ottawa, Ottawa, ON, Canada; ^10^ Department of Child and Adolescent Psychiatry, Charité—Universitätsmedizin, Berlin, Germany; ^11^ Department of Psychiatry, University of Campania “Luigi Vanvitelli”, Naples, Italy; ^12^ Division of Pediatric Gastroenterology and Nutrition, Department of Pediatrics, Massachusetts General Hospital for Children, Harvard Medical School, Boston, MA, United States; ^13^ Mucosal Immunology and Biology Research Center, Massachusetts General Hospital for Children, Boston, MA, United States

**Keywords:** eating disorders, artificial intelligence, data-driven approach, early identification, personalized treatment

## Abstract

**Introduction:**

Eating Disorders (EDs) affect individuals globally and are associated with significant physical and mental health challenges. However, access to adequate treatment is often hindered by societal stigma, limited awareness, and resource constraints.

**Methods:**

The project aims to utilize the power of Artificial Intelligence (AI), particularly Machine Learning (ML) and Deep Learning (DL), to improve EDs diagnosis and treatment. The Master Data Plan (MDP) will collect and analyze data from diverse sources, utilize AI algorithms for risk factor identificat io n, treatment planning, and relapse prediction, and provide a patient-facing chatbot for information and support. This platform will integrate patient data, support healthcare professionals, and empower patients, thereby enhancing care accessibility, personalizing treatment plans, and optimizing care pathways. Robust data governance measures will ensure ethical and secure data management.

**Results:**

Anticipated outcomes include enhanced care accessibility and efficiency, personalized treatment plans leading to improved patient outcomes, reduced waiting lists, heightened patient engagement, and increased awareness of EDs with improved resource allocation.

**Discussion:**

This project signifies a pivotal shift towards data-driven, patient-centered ED care in Italy. By integrat ing AI and promoting collaboration, it seeks to redefine mental healthcare standards and foster better well- being among individuals with EDs.

## Introduction

An Eating Disorder (ED) is a mental disorder characterized by abnormal eating behaviors that adversely affect a person’s physical or mental health and quality of life (1, 2). The reported prevalence of EDs in the general population has varied widely, ranging from 0.1% to 3.8% (3). Global incidence rates have increased from 3.4% calculated between 2000 and 2006 to 7.8% between 2013 and 2018 (4). The incidence of a first diagnosis of an ED has increased during the Covid- 19 pandemic; an overall excess of 15.3% was observed compared to 2019 (5). Individuals with EDs may also develop severe somatic complications that can increase the risk of suicide (6) and mortality rates. Anorexia Nervosa (AN) has among the highest mortality and suicide rates in mental health (2, 7). Over 3.3 million healthy life years are lost worldwide due to EDs each year, and many more are lost to disability due to medical and psychiatric complications (8). Suicide accounts for approximately 20% of non-natural deaths among people with ED (9). The criteria for EDs have evolved, with the DSM-5 (10) expanding the diagnostic categories to “feeding and eating disorders.” Binge Eating Disorder (BED) is now listed individually. Apart from AN, BN and BED, other disorders are classified as Other Specified Feeding and Eating Disorder (OSFED) and Unspecified Feeding and Eating Disorder (UFED). OSFED includes disorders like atypical AN, atypical BN, atypical BED, purging disorder, and night eating syndrome, which cause clinical suffering or social impairment but do not meet the specific criteria for AN, BN, or BED. UFED encompasses other eating disorders that cause clinical distress or social dysfunction but do not meet the criteria for any listed conditions.

Historically, EDs were thought to be more prevalent in Western countries and among women, influenced by cultural beliefs and attitudes (11). With industrialization and globalization, many regions have reported increasing incidence rates of EDs (12, 13). People of all ages, ethnicities, and socioeconomic conditions (14) can be affected by EDs, although adolescents and young adults are particularly at risk, and the mean age of onset is decreasing (15). All EDs are marked by frequent psychiatric and physical comorbidity (16), and impaired physical, social, and work functioning (6, 17). The etiopathogenesis of EDs is thought to be multifactorial, with models postulating the presence of predisposing factors [genetic vulnerability (18, 19)], temperamental traits, and childhood traumatic experiences (20), precipitating factors [the environmental context at the time of onset (21)], and maintaining factors [secondary aspects of the illness, such as brain adaptation induced by malnutritio n, social isolation, and changes in the environment (22)]. However, a clear understanding of this etiopathogenesis is currently lacking, although it would be essential to improve treatment effectiveness (23). Access to treatment for EDs is inadequate, with only 20-25% of individuals receiving professiona l consultation for their symptoms (24). Barriers to treatment access include stigma, lack of insight into the illness, shame, scarce availability of evidence-based interventions, and fragmented or underfunded health services (25, 26), which contribute to low recovery rates and frequent chronicity (27). In Italy, guidelines for the treatment of EDs have been licensed by the Ministry of Health and follow the most common international evidence-based treatment options (28). Despite these guidelines, Volpe et al. (29) revealed an important delay from the onset of ED symptoms to treatment and an unequal presence throughout the whole country of the three levels of care (outpatient, semi-residential, residentia l) identified as necessary by the same guidelines.

The Italian government’s National Action Plan for Mental Health (NAPMH) aims to improve mental healthcare, especially in response to the COVID-19 pandemic. The plan focuses on improving mental health services by creating specialized care pathways for different conditions and upgrading facilities like residential centers. It also emphasizes early intervention for eating disorders (EDs) by promoting collaboration between mental health services and emergency departments, and exploring the use of advanced artificial intelligence (AI) techniques for earlier ED detection. Overall, the NAPMH strives to provide a more effective and integrated system of mental healthcare for the Italian population.

In recent years, the integration of AI techniques, particularly Machine Learning (ML) and Deep Learning (DL), has shown promise in enhancing both diagnosis and treatment strategies for EDs. This section provides an overview of the methodologies employed in clinical practice, their outcomes, and emphasizes the critical importance of their application. In clinical settings, AI methodologies have been leveraged to facilitate early identification, improve diagnostic accuracy, and personalize treatment plans for individuals with EDs. Studies have demonstrated the efficacy of ML algorithms in analyzing diverse datasets, including patient demographics, clinical assessments, and neuroimaging data, to identify patterns indicative of EDs (30, 31). These algorithms utilize supervised learning techniques to classify individuals based on symptom profiles, enabling clinicians to intervene promptly and tailor intervent ions to specific patient needs (32). Furthermore, AI-driven approaches have revolutionized treatment paradigms by optimizing therapeutic interventions and monitoring patient progress over time. For instance, natural language processing (NLP) algorithms have been employed to analyze textual data from patient diaries, online forums, and social media platforms, providing valuable insights into ED symptomatology and treatment outcomes (33). By extracting linguistic patterns and semantic cues from these sources, NLP models can identify high-risk individuals, assess treatment adherence, and predict relapse probabilities with high accuracy (34).

In recent years, DL techniques, such as convolutional neural networks (CNNs) and recurrent neural networks (RNNs), have emerged as powerful tools for modeling complex relationships within ED datasets. DL models can process multimodal inputs, including images, videos, and audio recordings, to capture nuanced features relevant to ED pathology (35). For example, CNNs trained on neuroima ging data have demonstrated the ability to detect structural and functional abnormalities in brain regions implicated in EDs, offering insights into neurobiological mechanisms underlying disordered eating behaviors (36).

The integration of ML and DL methodologies into clinical practice is essential for several reasons. Firstly, these techniques enable the analysis of large-scale datasets encompassing diverse patient populatio ns, facilitating the discovery of novel biomarkers, risk factors, and treatment targets for EDs (37). Secondly, AI-driven models enhance diagnostic precision by accounting for the heterogeneity of EDs presentations across individuals, thus reducing delays, misdiagnosis and optimizing resource allocation within healthcare systems. In summary, ML predicts and identifies relevant predictive factors, especially during adolescence and young adulthood for early identification. Through the analysis of big data ML and DL play a crucial role in identifying optimal therapeutic strategies, monitoring individual progress, predicting ED status, devising personalized treatment plans, implementing ecological momentary interventions, and analyzing complex interactions from various data sources to improve prediction accuracy (38–40). However, the translation of these promises into practice faces hindrances, such as the distant integration of technology into clinical practice, a lack of authentic databases for data-intensive AI algorithms, and the detachment of model development and validation from fundamental clinical utility and ethical standards (41).

## Aims of the project

In this context, the Local Health Unit of Salerno’s Department of Mental Health has strategica lly implemented its planning in accordance with the guidelines outlined by the NAPMH. This initia t ive entails enhancing information systems through the integratio n of scientific evidence and research within the field of Mental Health. The overarching objective of this project is to enhance the therapeutic services available, addressing the surge in psychological distress resulting from the ongoing epidemic. The focus is on the Regional Residential Center for Eating Disorders ‘Mariconda’ in Salerno, an integral component of the Regional Network for Eating Disorders in the Campania Region. This concerted effort aims to strengthen the mental health support infrastructure and ensure an effective response to the escalating challenges posed by the current public health situation. This project therefore aims to achieve, among other objectives, the following strategic/operational goals:

- Updated mapping of all services in the territorial network;
- Access functionality to the care-taking archives, providing information on active health services and the status of the individual within their own care setting;
- Management and monitoring dashboard;
- System for receiving and sending notifications integrated with the transitions dashboard;
- Privacy and data security;
- Reporting and flow generation

## Methods

To achieve these objectives, we have developed, in collaboration with IT.Svil s.r.l., a data-driven ecosystem called the Master Data Platform (MDP). This approach will enable us to utilize an objective decision-making algorithm that is “data-driven” rather than relying on subjective judgments prone to operator-dependent errors. Furthermore, this multimedia platform allows us to access and share real-time patient data. Not only does this platform aim to collect, store, organize, and process valuable data securely across the hospital and university network of the Campania Region regarding EDs, but it also aims to aid through AI to physicians involved in care pathways across different healthcare settings. MDP is designed to be an important resource for patients and their families.

For individuals struggling with AN, MDP will go beyond mere calorie counting, analyzing data on their social anxieties, underlying depression, and exercise preferences, creating a personalized therapy plan that addresses these intertwined factors.The chatbot feature will provide education on healthy eating, body image distortion, and the importance of building a positive relationship with food. Regarding people suffering from BN, MDP will recognize the complex cycle of binging and purging, focusing on developing coping mechanisms for emotional regulation and reducing self-critical thoughts.The platform’s AI capabilities will help identify triggers and suggest tailor-made mindfulness and relaxation techniques. Additionally, the chatbot will offer insights on managing binge urges and developing healthy purging alternatives. For individuals experiencing frequent episodes of binge eating, MDP will prioritize identifying and addressing underlying emotional distress.The platform will use data to personalize nutritional counseling, focusing on creating balanced meal plans and establishing healthy eating patterns.The chatbot will educate on mindful eating practices and provides support for overcoming emotional triggers associated with binging. MDP will go beyond leveraging data and technology by fostering active patient participation. By giving patients and families access to valuable information and resources, MDP will empower them to become active partners in their recovery journey. This collaborative approach enhances engagement, motivation, and the likelihood of achieving sustainable, long-term recovery. MDP recognizes the vital role of human connection in the recovery process, facilitating open communication between patients, families, and healthcare professionals. This supportive network is crucial for promoting healing and preventing relapse. Understanding that EDs are complex, MDP integrates data-driven personalization with a holistic approach that addresses both physical and mental health needs.

The system will be able to integrate with various regional and corporate healthcare systems of interest (e.g., patient registries, hospital admission management systems, etc.).

MDP is also designed to minimize treatment shedding and provide comprehensive coping options through a scalable, flexible architecture that prioritizes interoperability with various healthcare systems.

Interoperability will be integrated with healthcare systems for centralized, accessible patient data, ensuring coordinated care. The Real-Time Data Analysis will allow immediate treatment adjustments based on current patient status. The Personalized Treatment Plans will see a data-driven algorithm to tailor treatment plans to individual needs, enhancing relevance and adherence. The Patient and Family Engagement will provide valuable information and resources via an interactive chatbot, fostering active participation and support. The coping options provided by MDP will include educational resources that guide patients on healthy eating, exercise, coping strategies, and crisis protocols. It facilitates support networks by enhancing communication between patients, families, and healthcare professionals, providing essential emotional and psychological support. MDP takes a holistic approach by integrating mental and physical health needs. It also uses AI-assisted guidance to deliver evidence-based recommendations tailored to individual patient needs. Additionally, the platform includes real-time monitoring and alerts to track patient progress and ensure timely interventions.

**Figure 1:**
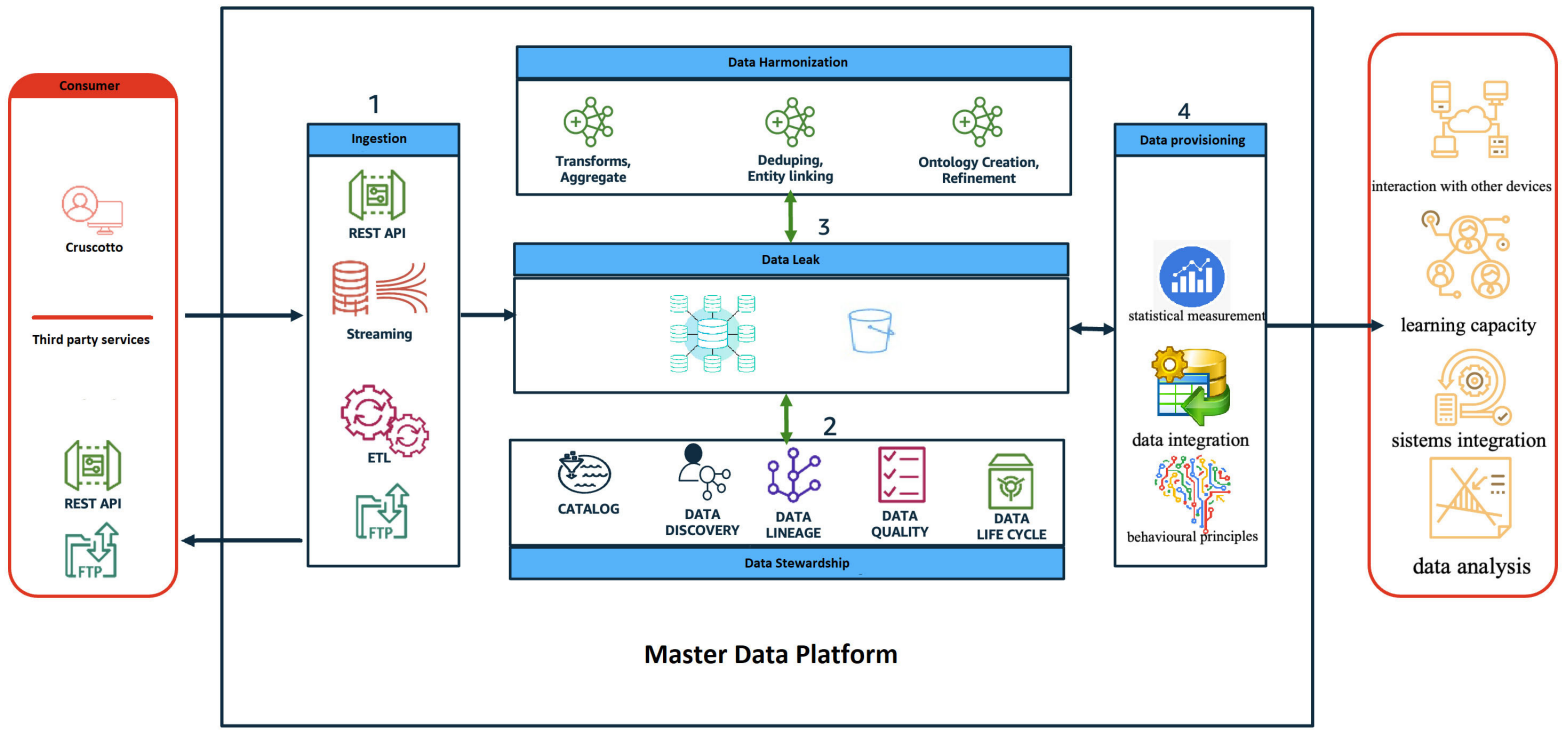
The diagram provides a high-level view of the components and how they interact through the different phases of the data life cycle.

For each implemented system, there will be a defined entry point through specific APIs, each of which is governed by specific data control policies (governance) and data protection measures. To ensure interoperability via APIs, recognized healthcare standards such as HL7/FHIR, HL7, HL7/CDA2, etc., will be used. Furthermore, from a security perspective, secure communication protocols such as HTTP over SSL, SFTP, SCP, VPN, etc., will be utilized (see **Figure 1**).

The role and behavior of each application layer is described below:

- Consumer: represents all the users of the MDP system both as an input system (i.e. health workers, patients/caregivers, etc) and as an output system (i.e. regional operators, patients/caregivers, health workers, etc);
- Ingestion: represents the frontier layer of MDP and has the role of servicing all the input requests coming from the consumer systems.
- Data Harmonisation: in this layer the input data are “harmonised, i.e., all the data cleaning and/or augmentation techniques/procedures are applied where necessary.
- Data stewardship: has the task of ‘administering’ the data, in terms of quality, privacy and security.
- Data leak: represents the application layer in charge of data storage.
- Data provisioning: provides tools/routines/programmes with the objective of transforming data to make it information, for example:
  ➔ ML algorithms to perform predictive analysis;
  ➔ Generate reporting (BI);
  ➔ Producing data flows, e.g. in CSV format (e.g. health flows)

The platform endeavors to construct an IT infrastructure that embraces a comprehensive approach, not merely from a functional standpoint, but also in terms of user experience. It comprises various components, each with its own objectives, yet collectively contributing to a unified purpose. For instance, leveraging technologies like mobile devices empowers patients to actively engage in their treatment journey, revolutionizing therapeutic approaches. Simultaneously, healthcare professionals gain access to control tools fostering continuous and direct patient interaction.

These individual components converge to amass data, forming a ‘source of truth’ utilized for multifario us purposes, including:

1. Evaluating digital, environmental, and blood biomarkers as predictive indicators of EDs pathology relapse or remission.
2. Enhancing digital technologies for predicting psychopathology severity and evaluating treatment efficacy.
3. Employing technologies to differentiate subjects and tailor personalized approaches.

In terms of technological solutions, the envisaged integrated platform incorporates sensors and smart devices, proficient in collecting, analyzing, and interpreting data pertaining to digital biomarker clusters.

The design ethos, encompassing both hardware and software, adheres to principles of openness, integrability, and scalability.

To encapsulate, the platform encompasses:

1. Web and mobile applications capable of integrating third-party smart devices (e.g., smartwatches), gathering physiological (heart rate, saturation, etc.) and behavioral data (movement, sleep patterns, voice, etc.).
2. A data ingestion module implementing policies for filtering, transforming, and normalizing acquired data.
3. A virtual HUB (concentrator), a cloud-native software component ensuring data integrity.
4. Implementation of artificial intelligence decision-support algorithms utilizing neural network architectures for Deep Learning to optimize decision- making models and therapeutic governance.
5. Deployment of value-added applications for data analysis, structured according to standards like the International Patient Summary (IPS), rather than conventional time-series dashboards.

## Results

This project aims, in a futuristic manner, to develop an MDP platform specifically designed for the management and treatment of eating disorders for the first time in a territorial psychiatric care context. The objectives we aim to achieve include creating a more accessible and welcoming environment for people with ED, thanks to a change in perspective that allows patients to be active participants in dialogues with both the program’s chatbot and various operators. Additionally, a better customization of treatments is expected, as each patient’s data will be available to attending physicians, replacing the paper medical record. Moreover, the program will take into account a range of psychosocial variables that can improve treatment adherence, quality of life, and hopefully patient satisfaction with the service. Another important outcome expected is more effic ie nt management of waiting lists, minimizing delays in accessing care. This will be made possible by a streamlined and responsive system that will ensure timely attention and support to those in need. Another key result is the adaptation of mental health center care plans to individua l needs and the optimal alignment of care intensity with the specific needs of each case.

This project seeks to elevate the quality of mental healthcare for patients with eating disorders. Through meticulous evaluations of admissions and transfers between facilities, we can guarantee care that prioritizes the individual patient’s needs. Robust data governance measures will be established to safeguard sensitive information and set a benchmark for responsible data management within mental health. Integration of artificial intelligence (AI) into clinical processes promises enhanced diagnostic accuracy and overall efficiency. The project strives to achieve high-quality, technology-driven care that empowers both physicians and patients. This collaborative approach will enable patients to feel actively involved in their recovery journey. Additionally, the project aims to raise awareness regarding the significance of implementing a comprehensive range of care options, encompassing outpatient, semi- residential, and residential programs, to best serve patients with eating disorders across the region.

The development of such a platform and the optimiza t ion of care, as well as the involvement and increased ease of data sharing among multiple facilities located in different parts of the territory, would promote expansion of the centralized Hub and consequently a dynamic bidirectional flow of information and resources, facilitating collaboration and resource sharing among healthcare providers. The expected outcome of the departmental Hub is the effective monitoring of user clusters in all Spoke facilities. Regular reports disseminated to the Spoke will mean transparency and a cohesive approach to mental health care.

## Discussion

The project aims to create an MDP platform for managing EDs within territorial psychiatric care. Its benefits include fostering a welcoming environment that enhances patient engagement and satisfaction. Digital medical records will facilitate personalized treatments, strong data governance measures will protect sensitive information, while a collaborative approach empowers patients and healthcare providers. A centralized Hub will promote dynamic information flow, resource sharing and systematic monitoring with regular reporting to maintain cohesive mental health care. However, there are still many challenges: managing digital patient data demands stringent cybersecurity to prevent breach; developing and integrating the platform into existing systems can be complex and resource-intensive. Ensuring access to technology for all patients and facilities, especially in under-resourced areas, could be difficult. There might be resistance to transitioning from traditional methods to a technology-driven approach, necessitating change management efforts. The project’s costs, including setup, maintenance, and upgrades, require sustainable funding. Seamless integration with current health records and care systems may present technical challenges, and staff will need extensive training. Over-reliance on digital solutions could lead to vulnerabilities in case of technology failures or cyberattacks. Ethical considerations around consent, data ownership, and potential misuse of AI and digital data also need to be addressed. The transformative potential of the project to improve the treatment of EDs through innovative, patient-centered technology will overcome these challenges, offering a promising pathway to more effective and integrated mental health services.

## Author contributions

FM: Conceptualization, Methodology, Supervision, Visualization, Writing – original draft, Writing – review & editing. AV: Conceptualization, Methodology, Supervision, Validation, Writing – original draft, Writing – review & editing. MP: Investigation, Supervision, Visualization, Writing – review & editing. CP: Conceptualization, Project administration, Resources, Writing – review & editing. CM: Methodology, Project administration, Visualization, Writing – review & editing. LS: Supervision, Validation, Visualization, Writing – review & editing. AM: Writing – review & editing. FF: Writing – review & editing. GP: Conceptualization, Data curation, Methodology, Writing – review & editing. SL: Writing – review & editing. EF: Visualization, Writing – review & editing. SP: Visualization, Writing – review & editing. VM: Supervision, Validation, Writing – review & editing. MS: Supervision, Validation, Visualization, Writing – review & editing. AMM: Supervision, Validation, Visualization, Writing – review & editing. AF: Supervision, Validation, Visualization, Writing – review & editing. GC: Resources, Supervision, Visualization, Writing – review & editing.
